# Racial/Ethnic Disparities: Discrimination’s Impact on Health-Related Quality of Life—An All of Us Cancer Survivors’ Cross-sectional Study

**DOI:** 10.1007/s40615-024-02006-z

**Published:** 2024-04-23

**Authors:** Angel Arizpe, Carol Y. Ochoa-Dominguez, Stephanie Navarro, Sue E. Kim, Katelyn Queen, Trevor A. Pickering, Albert J. Farias

**Affiliations:** 1 Keck School of Medicine of the University of Southern California, 1845 N. Soto St., Suite 318B, Los Angeles, CA 90032 USA; 2 University of California, San Diego, San Diego, CA USA

**Keywords:** Discrimination in medical settings, All of Us, Perceived discrimination, Cancer survivor, Health-related quality of life

## Abstract

**Background:**

Discrimination is associated with worse mental and physical health outcomes. However, the associations among cancer survivors are limited.

**Objective:**

We examined whether discrimination is associated with HRQoL and whether adjusting for it reduces racial/ethnic disparities in HRQoL among cancer survivors.

**Methods:**

Cross-sectional data from adult cancer survivors who completed surveys on discrimination in the medical settings (DMS), everyday perceived discrimination (PD), and HRQoL in the “All of Us” Program from 2018 to 2022 were assessed. We created a binary indicator for fair-to-poor vs. good-to-excellent physical health and mental health. PD and DMS scores were a continuous measure with higher scores reflecting more discrimination. Multivariable logistic regression models tested whether DMS and PD are associated with HRQoL and whether they differently affect the association between race/ethnicity and HRQoL.

**Results:**

The sample (*N* = 16,664) of cancer survivors was predominantly White (86%) and female (59%), with a median age of 69. Every 5-unit increase in DMS and PD scores was associated with greater odds of fair-to-poor physical health (DMS: OR [95%CI] = 1.66 [1.55, 1.77], PD: 1.33 [1.27, 1.40]) and mental health (DMS: 1.57 [1.47, 1.69], PD: 1.33 [1.27, 1.39]). After adjusting for DMS or PD, Black and Hispanic survivors had a decreased likelihood of fair-to-poor physical health and mental health (decrease estimate range: − 6 to − 30%) compared to White survivors. This effect was greater for Black survivors when adjusting for PD, as the odds of fair-to-poor mental health compared to White survivors were no longer statistically significant (1.78 [1.32, 2.34] vs 1.22 [0.90, 1.64]).

**Conclusion:**

Experiences of discrimination are associated with lower HRQoL and reducing it may mitigate racial/ethnic disparities in HRQoL.

**Supplementary Information:**

The online version contains supplementary material available at 10.1007/s40615-024-02006-z.

## Background

By 2026, there will be over 20 million cancer survivors In the United States (US) [1, 2]. While the increase in those surviving cancer is a result of advancement in cancer treatment, evidence suggests that cancer and cancer treatment negatively affect mental (e.g., stress, depression symptomology, anxiety) and physical (e.g., diabetes, chronic pain, cardiovascular problems) health [3, 4]. These adverse effects from diagnosis and treatment can interfere with activities of daily living, social interactions, and overall autonomy, diminishing survivors’ HRQoL [3–6]. Even more alarming is that racial/ethnic disparities in HRQoL in US populations are consistent, with non-Hispanic Black and Hispanic individuals experiencing a greater likelihood of reporting fair-to-poor HRQoL compared to their non-Hispanic White counterparts [7–12].

The drivers of racial/ethnic disparities in HRQoL include socioeconomic (SES) factors such as income and employment, and access and utilization to medical services [13]. Additionally, treatment, health behaviors, cultural aspects, social support, and geographical location are factors that can influence the HRQoL of individuals [14, 15]. Perhaps the most important and critically understudied reason for the disparities in HRQoL may be with experience of discrimination, particularly in medical settings.

Discrimination is a critical social determinant of health [16] that can be described as a stress-inducing factor, including hostile attitudes or unjust actions directed towards individuals belonging to specific groups [17]. The negative associations of discrimination with mental and physical health via multiple mechanisms are well-known [6, 18, 19]. For example, discrimination can lead to worse health outcomes via decreased access to care, worse quality of care, and lower utilization of health services [20]. Discrimination can also directly increase chronic stress, reducing the body’s biological mechanisms to regulate stress and its downstream effects leading to adverse health outcomes [21] including cardiovascular problems, chronic pain, decreased self-reported overall health, anxiety, and depression [22]. Furthermore, discrimination restricts access to essential goods and services via structural discrimination as policies in practice may have a differential effect on racial/ethnic minoritized communities, hindering individuals from receiving necessary care [23]. Racial/ethnic minorities who experience discriminatory events report poorer quality of care and healthcare access compared to their Non-Hispanic White counterparts [24], but few studies have investigated discrimination and health-related quality of life (HRQoL) defined as the global physical and global mental health in cancer survivor populations from multiple cancer types [6, 25]. Thus, this study contributes to the literature by exploring this association using the All of Us dataset, which includes participants from multiple geographical and diverse SES areas in the US and a range of cancer types given that previous findings were among breast and ovarian cancer survivors.

Discrimination in the medical setting (DMS) can be particularly harmful to cancer survivors as it may lead individuals to delay needed care. DMS refers to unfavorable actions, mistreatment, and behaviors towards an individual or group, stemming from preconceived beliefs and opinions [26] while receiving healthcare services. Racial/ethnic minorities are more likely to encounter discrimination in medical settings compared to their non-Hispanic White counterparts [27]. Additionally, in a study among limited-resourced cancer survivors, a majority (58%) reported experiencing discrimination from their healthcare providers (e.g., doctors, nurses) based on their socioeconomic, demographic, and clinical factors (i.e., disease status and comorbidities), suggesting that cancer survivors may experience discrimination throughout all healthcare levels [28, 29]. Discriminatory experiences in healthcare can increase avoidance of needed care, further hindering individuals’ HRQoL [23, 30–32]. To our knowledge, no studies have assessed the relationship of discrimination in the medical setting with HRQoL among cancer survivors, which is important as they require life-long surveillance [33, 34] to prevent, monitor, and manage chronic health conditions, sequelae of cancer treatment, and cancer reoccurrence. [33]

To fill this gap in the literature, this study used a large and geographically diverse sample of cancer survivors from across the U.S., by leveraging data from the All of Us Research Program, to identify whether DMS or everyday perceived discrimination (PD) is associated with HRQoL and whether they independently contribute to racial/ethnic disparities in HRQoL.

## Methods

### Data Collection and Sample

Cross-sectional survey data collected from May 2018 to July 2022 from “All of Us” was analyzed. The All of Us program is open to anyone aged 18 and older in the US. Participants signed a consent form following the Declaration of Helsinki for data collection. Data used for this study were de-identified and made available to approved researchers. The All of Us program was approved by the National Institutes of Health (NIH) Institutional Review Board (IRB).

**Fig. 1:**
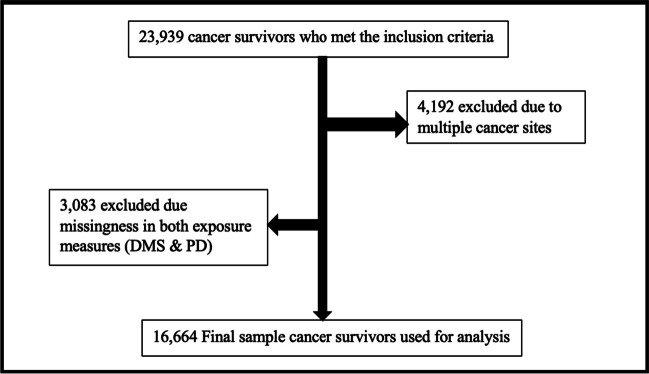
Decision tree outlining the cohort’s inclusion and exclusion criteria

Inclusion criteria for our cohort included participants who were ever told by their healthcare provider that they have cancer and had a recorded survey completion date in the Social Determinant of Health (SDoH) survey. We excluded participants with missing data on self-reported discrimination in the medical setting and everyday perceived discrimination and those with multiple cancer sites (Fig. 1).

### Measures

#### Demographics and Covariates

Demographic characteristics included in our study were age (at survey completion), sex (male vs. female), race/ethnicity (non-Hispanic White vs. categories: non-Hispanic Asian, non-Hispanic Black, Hispanic, and, Other [includes more than one race, another race, and none of these]), marital status (married [includes living with a partner] vs single [includes single, divorced, widowed, and separated]), current cancer treatment (yes vs. no), cancer type, and socioeconomic barrier index (SES): five SES factors (education [≤ high school], income [≤ 35 K], insurance [none], housing [rent/other], and employment status [unemployed]) were dichotomized to create a composite measure as detailed in a previous study ranging from 0 to 5, that were truncated to 3 + due to sparsity. Higher scores indicate higher socioeconomic barriers. [35]

### Exposure

#### Discrimination in the Medical Settings (DMS)

The 7-item DMS [36] (Cronbach’s *α* = 0.89) is an adapted scale from the Everyday Discrimination Scale (EDS) [37], which assesses the participants’ prior treatment experiences while getting healthcare services. Example items include “You feel like a doctor or nurse is not listening to what you were saying,” “A doctor or nurse acts as if he or she thinks you are not smart.” Responses were measured on a 5-point Likert scale. A 5-unit scaled continuous variable was created by taking the mean of these survey items with higher scores representing experiencing higher discrimination in medical settings (Supplementary Table 1).

#### Everyday Perceived Discrimination (PD)

Perceived discrimination was assessed using the EDS questionnaire [37] (Cronbach’s *α* = 0.90). This scale asked participants nine questions about the frequency with which they experienced discrimination in their day-to-day lives. Example items include “You are called names or insulted” and “You are threatened or harassed.” Responses were measured on a 5-point Likert scale. A 5-unit scaled score variable was created where higher scores indicate higher experiences of perceived discrimination (Supplementary Table 2).

### Outcome

#### Health-Related Quality of Life (HRQoL)

HRQoL was assessed using the Patient-reported Outcomes Measurement Information System (PROMIS-10) Global Health [38] scale. PROMIS has been utilized across different chronic health conditions [39] to assess physical, mental, and social domains of health from the patient’s view. The physical health (Cronbach’s *α* = 0.71) and mental health (Cronbach’s *α* = 0.76) constructs were created based on four items each from the standardized PROMIS-10 score. The physical health items measured the overall physical health, activities, pain, and fatigue assessment, whereas mental health items assessed the overall quality of life, mental and emotional problems, and social activities and satisfaction. Studies suggest that fair-to-poor PROMIS-10 scores in mental health and physical health are positively associated with increased healthcare utilization [28, 40]. To assess HRQoL using the physical health and mental health, we created two indicator variables from their respective T-scores (physical health range 16.2–67.2 vs mental health range 21.2–67.6). Using the PROMIS T-score cut points, those with values < 42 in physical health and values < 40 in mental health were described as having fair-to-poor health status, any values ≥ 42 or ≥ 40, respectively, were considered as good-to-excellent physical health and mental health status [40, 41] (Supplementary Table 3).

### Statistical Methods

Descriptive statistics used chi-square or Mann–Whitney *U* tests to determine the association of all the variables with the exposure (EDS and DMS) and outcome (physical health and mental health), separately. Multivariable logistic regression models assessed whether DMS or PD were associated with HRQoL. To assess whether DMS or PD independently attenuate the racial/ethnic disparities in HRQoL, we conducted unadjusted (model 1) and adjusted models (model 2 [adjusted by DMS or PD]; model 3 [adjusted by DMS or PD + covariates]). Covariates included in multivariable models (model 3) were age, sex, nativity, SES barriers index, cancer type, and treatment status. Percent changes were calculated from the unadjusted (model 1) to the adjusted (model 2) models to quantify the level of attenuation once DMS or PD were included in the model. All statistical analyses were performed using R Jupyter Notebooks accessed via the “All of Us” workbench and using a significance level at alpha > 0.05. Odds ratios (ORs) with 95% confidence intervals (CI) and *p*-values were reported.

## Results

Our final analytical sample consisted of 16,664 cancer survivors with a median age of 69 (interquartile range [IQR (Q1, Q3)] = 59.9, 74.6) years. Most participants identified as non-Hispanic White as their race/ethnicity (86%) and reported their biological sex as female (59%). The majority of cancer survivors reported being married (67%), US-born (91%), and having good-to-excellent physical (79%) and mental health (59%) (Table 1). Cancer survivors who self-identified as non-Hispanic Black, experienced 3 or more SES barriers, or those who reported fair-to-poor physical and mental health had a higher median DMS score. A similar pattern was observed for PD scores (Table 1).

**Table 1:** Sample descriptive characteristics of cancer survivors’ discrimination (in the medical setting and overall perceived discrimination) scores and their bivariate associations (*N* = 16,664)

Variables	Total	DMS score Median (IQR [Q1, Q3])	*p*	PD score Median (IQR [Q1, Q3])	*p*
Sex			< 0.001		< 0.001
Male	6316 (37.9%)	0.4 (0.0, 1.0)		0.6 (0.0, 1.6)	
Female	9899 (59.4%)	0.6 (0.0, 1.2)		1.0 (0.2, 1.6)	
Missing	449 (2.7%)	0.4 (0.0, 1.2)		0.8 (0.0, 1.8)	
Race/ethnicity			0.03		< 0.001
White	14,399 (86.4%)	0.4 (0.0, 1.0)		0.8 (0.0, 1.6)	
Hispanic	801 (4.8%)	0.6 (0.0, 1.4)		1.2 (0.2, 2.4)	
Black	572 (3.4%)	0.8 (0.0, 1.4)		1.5 (0.6, 2.6)	
Asian	157 (0.9%)	0.6 (0.0, 1.2)		1.2 (0.4, 2.2)	
Other	113 (0.7%)	0.8 (0.2, 1.6)		1.8 (0.8, 2.6)	
Missing	622 (3.7%)	0.4 (0.0, 1.2)		0.8 (0.0, 1.8)	
Age			< 0.001		< 0.001
Median (IQR) or correlation	68.6 (59.9, 74.6)	− 0.17		− 0.28	
Income			< 0.001		< 0.001
Lowest quintile	3606 (21.6%)	0.6 (0.0, 1.4)		1.2 (0.2, 2.0)	
Rest	13,058 (78.4%)	0.4 (0.0, 1.0)		0.8 (0.0, 1.6)	
Marital status			< 0.001		< 0.001
Married	11,110 (66.7%)	0.4 (0.0, 1.0)		0.8 (0.0, 1.6)	
Single	5088 (30.5%)	0.6 (0.0, 1.2)		1.0 (0.2, 2.0)	
Missing	466 (2.8%)	0.6 (0.0, 1.2)		0.9 (0.2, 1.8)	
Education status			0.32		0.07
> High school	14,955 (89.7%)	0.4 (0.0, 1.2)		0.8 (0.0, 1.8)	
≤ High school	1253 (7.5%)	0.4 (0.0, 1.2)		0.8 (0.0, 2.0)	
Missing	456 (2.7%)	0.6 (0.0, 1.2)		0.8 (0.0, 1.8)	
Insurance status			< 0.001		< 0.001
Uninsured	16,063 (96.4%)	0.4 (0.0, 1.2)		0.8 (0.0, 1.8)	
Insured	155 (0.9%)	0.8 (0.2, 1.6)		1.4 (0.0, 2.6)	
Missing	446 (2.7%)	0.6 (0.0, 1.4)		0.9 (0.0, 1.8)	
Nativity			0.05		0.29
USA	15,226 (91.4%)	0.4 (0.0, 1.2)		0.8 (0.0, 1.8)	
Foreign	1022 (6.1%)	0.4 (0.0, 1.0)		0.8 (0.0, 1.8)	
Missing	416 (2.5%)	0.6 (0.0, 1.2)		0.8 (0.0, 1.8)	
Housing status			< 0.001		< 0.001
Own	13,059 (78.4%)	0.4 (0.0, 1.0)		0.8 (0.0, 1.6)	
Rent/other arrangement	3098 (18.6%)	0.8(0.0,1.4)		1.4 (0.4, 2.0)	
Missing	507 (3.0%)	0.6 (0.0, 1.4)		0.8 (0.0, 1.8)	
Employment status			0.07		< 0.001
Employed	14,846 (89.1%)	0.4 (0.0, 1.0)		0.8 (0.0, 1.6)	
Unemployed	1356 (8.1%)	1.0 (0.2, 1.8)		1.6 (0.4, 2.8)	
Missing	462 (2.8%)	0.4 (0.0, 1.2)		0.8 (0.0, 1.8)	
SES barriers			< 0.001		< 0.001
0 (no barriers)	11,426 (68.6%)	0.4 (0.0, 1.0)		0.8 (0.0, 1.6)	
1	3284 (19.7%)	0.6 (0.0, 1.2)		1.0 (0.2, 2.0)	
2	1208 (7.2%)	0.8 (0.0, 1.6)		1.4 (0.4, 2.4)	
3	746 (4.5%)	1.0 (0.2, 1.8)		1.8 (0.6, 3.2)	
Global Physical Health			< 0.001		< 0.001
Good-to-excellent	13,121 (78.7%)	0.8 (0.0, 1.0)		0.8 (0.0, 1.6)	
Fair-to-poor	2332 (14.0%)	1.0 (0.4, 1.8)		1.6 (0.6, 2.8)	
Missing	1211 (7.3%)	0.6 (0.0, 1.4)		1.0 (0.2, 2.0)	
Global mental health			< 0.001		< 0.001
High (good-to-excellent)	9799 (58.8%)	0.6 (0.0, 1.2)		1.0 (0.2, 1.6)	
Low (fair-to-poor)	1157 (6.9%)	1.2 (0.4, 1.8)		1.8 (0.8, 3.0)	
Missing	5708 (34.3%)	0.2 (0.0, 0.8)		0.4 (0.0, 1.4)	
Active treatment			0.15		0.86
No	8450 (74.0%)	0.4 (0.0, 1.2)		0.8 (0.0, 1.8)	
Yes	2933 (25.7%)	0.4 (0.0, 1.2)		0.8 (0.0, 1.8)	
Missing	43 (0.4%)	0.2 (0.0, 1.0)		0.8 (0.0, 1.6)	

*Notes*: *SES*, socioeconomic; *IQR*, interquartile range; *Q1*, quantile 0.25; *Q3*, quantile 0.75

*p*-values were obtained using Spearman’s correlation, Mann–Whitney, or Kruskal–Wallis tests

Married includes living with partner, single includes divorced, widowed, and separated

Regarding HRQoL, cancer survivors reporting fair-to-poor physical health (*n* = 1211), females accounted for a higher proportion (10.2%) compared to males (5.8%). Additionally, non-Hispanic Black (16.9%) and Hispanic (16.9%) cancer survivors had a higher proportion of individuals with fair-to-poor physical health compared to non-Hispanic White cancer survivors (< 10%), non-Hispanic Asian (< 15%), and Other (< 20%) race/ethnicity cancer survivors. This group was also characterized by being younger (61.8 years vs 69.4 [good-to-excellent]), single (12.9%) vs married (6.5%), foreign-born (10.5%) vs US-born (8.3%) and having 3 + SES barriers (46.7%) vs all other SES barrier levels (0 = 4.2%, 1 = 11.7%, 2 = 26.1%).

Similarly, a higher proportion of Hispanic (18.2%) cancer survivors vs. all other race/ethnicity categories (non-Hispanic White < 10%, non-Hispanic Black 16.3%, non-Hispanic Asian < 10%, Other < 20%) was observed for fair-to-poor mental health (*n* = 1157). Moreover, those who reported 3 + SES barriers (39%) vs. all other SES barrier levels (0 = 5.6%, 1 = 12.9%, 2 = 27.3%) exhibited a higher prevalence of fair-to-poor mental health, along with those who were, uninsured (27%) vs insured (10.3%), and those belonging to the lowest income quintile (22.1%) vs all rest of income quintiles (7%) (Table 2).

**Table 2:** Sample descriptive characteristics and their bivariate association with health-related quality of life (physical and mental health)

	Global physical health			Global mental health		
Variables	Good-to-excellent (*N* = 13,121) *n* (%)	Fair-to-poor (*N* = 1211) *n* (%)	*p*	Good-to-excellent (*N* = 9799) *n* (%)	Fair-to-poor (*N* = 1157) *n* (%)	*p*
Sex			***			0.12
Male	5246 (94.2)	322 (5.8)		3376 (90.0)	474 (10.0)	
Female	7545 (89.8)	858 (10.2)		6177 (89.3)	742 (10.7)	
Missing	330 (91.4)	31 (8.6)		246 (85.7)	41 (14.3)	
Race/ethnicity			***			***
White	> 11,500 (> 90)	< 1000 (< 10)		> 8400 (> 85)	< 950 (< 10)	
Hispanic	523 (83.1)	106 (16.9)		468 (81.8)	104 (18.2)	
Black	350 (83.1)	71 (16.9)		308 (83.7)	60 (16.3)	
Asian	> 100 (> 85)	≤ 20 (< 15)		> 90 (> 85)	≤ 20 (< 10)	
Other	> 50 (> 75)	≤ 20 (< 20)		> 50 (> 80)	≤ 20 (< 20)	
Missing	456 (89.4)	54 (10.6)		362 (89.6)	42 (10.1)	
Age			***			***
Median (IQR [Q1, Q3)])	69.4 [61.4, 74.6]	61.8 [51.7, 70.2]		67.6 [58.7, 73.7]	61.4 [51.4, 69.5]	
Income			***			***
Lowest quintile	2233 (80.3)	548 (19.7)		2004 (77.9)	568 (22.1)	
Rest	10,888 (94.3)	663 (5.7)		7795 (93.0)	589 (7.0)	
Marital status			***			***
Married	9119(93.5)	634 (6.5)		6682 (92.5)	542 (7.5)	
Single	3661 (87.1)	541 (12.9)		2852 (83.1)	579 (16.9)	
Missing	341 (90.5)	36 (9.5)		265 (88.0)	36 (12.0)	
Education status			***			***
> high school	12,069 (92.6)	966 (7.4)		8846 (90.4)	935 (9.6)	
≤ high school	730 (78.3)	202 (21.7)		686 (78.4)	189 (21.6)	
Missing	322 (88.2)	43 (11.8)		267 (89.0)	33 (11.0)	
Insurance status			***			***
Uninsured	85 (75.9)	27 (24.1)		81 (73.0)	30 (27.0)	
Insured	12,722 (91.7)	1144 (8.3)		9477 (89.7)	1088 (10.3)	
Missing	314 (88.7)	40 (11.3)		241 (86.1)	39 (13.9)	
Nativity			0.18			0.23
USA	12,043 (91.7)	1093 (8.3)		8967 (89.6)	1038 (10.4)	
Foreign	769 (89.5)	90 (10.5)		586 (87.3)	85 (12.7)	
Missing	309 (91.7)	28 (8.3)		246 (87.9)	34 (12.1)	
Housing status			***			***
Own	10,822 (94.0)	688 (6.0)		7792 (92.6)	623 (7.4)	
Rent/other arrangement	1939 (80.6)	472 (19.4)		1726 (78.1)	484 (21.9)	
Missing	343 (87.1)	51 (12.9)		281 (84.9)	50 (15.1)	
Employment status			***			***
Employed	12,253 (94.0)	783 (6.0)		8870 (92.0)	767 (8.0)	
Unemployed	538 (57.9)	391 (42.1)		672 (65.8)	350 (34.2)	
Missing	330 (89.9)	37 (10.1)		257 (86.5)	40 (13.5)	
SES barriers			***			***
0 (no barriers)	9787 (95.8)	434 (4.2)		6821 (94.4)	403 (5.6)	
1	2420 (88.3)	321 (11.7)		1995 (87.1)	295 (12.9)	
2	658 (73.9)	232 (26.1)		642 (72.7)	241 (27.3)	
3	256 (53.3)	224 (46.7)		341 (61.0)	218 (39.0)	
Active treatment			***			0.66
No	> 9700 (> 90)	> 800 (> 5)		> 7100 (> 85)	> 800 (> 5)	
Yes	3300 (90.0)	367 (10.0)		2613 (89.5)	306 (10.5)	
Missing	> 30 (> 80)	≤ 20 (< 20)		> 25 (> 100)	≤ 20 (< 10)	

*Notes*: *SES*, socioeconomic, married includes living with a partner, single includes divorced, widowed, and separated

Chi-square or Fisher tests were performed to obtain *p*-values (*p*)

Significant *p*-values *** < 0.001, ** < 0.01, * < 0.05

Per “All of Us” data use agreement policy, groups < 20 participants are shown as ≤ 20 (%) with a corresponding > (%) category to prevent deriving counts < 20 from other values

No all percentages equal to 100

### Discrimination and HRQoL

Results of multivariable models showed that a 5-unit increase in DMS score was associated with a 66% (OR = 1.66, 95% CI:1.55, 1.77) and 57% (OR = 1.57, 95% CI:1.47, 1.69) greater likelihoods of reporting fair-to-poor physical health and mental health, respectively (Table 3). Similar results were observed for PD scores where a 5-unit increase was significantly associated with a 33% [physical health: (OR = 1.33, 95% CI: 1.27, 1.40); mental health: (OR = 1.33, 95% CI: 1.27, 1.39)] greater likelihood of reporting fair-to-poor physical health and mental health (Table 3).

**Table 3:** Multivariable regression analysis of discrimination and health-related quality of life of cancer survivors from the All of Us Research Cohort

	Fair-to-poor Physical health (*n* = 13,555)	Fair-to-poor Mental health (*n* = 10,322)
	OR (95%CI)	OR (95%CI)
Discrimination in medical settings (DMS)	1.66 (1.55–1.77)***	1.57 (1.47–1.69)***
Perceived discrimination (PD)	1.33 (1.27–1.40)***	1.33 (1.27–1.39)***

*Notes*: Models adjusted for race/ethnicity, sex, age, marital status, active cancer treatment, SES barriers, cancer type, and nativity

*CI*, confidence interval, significant *p*-values *** < 0.001, ** < 0.01, * < 0.05

### Race/Ethnicity and HRQoL (Model 1)

Before controlling for DMS or PD and other confounding factors, there was not a statistically significant difference in the likelihood of reporting fair-to-poor physical health between Asian and White cancer survivors OR = 1.45 (95%CI: 0.79, 2.44). However, cancer survivors who identified as non-Hispanic Black and Hispanic, were 2.48 (1.89, 3.20), and 2.47 (1.98, 3.07) higher odds of reporting fair-to-poor physical health compared to non-Hispanic White cancer survivors, respectively. Non-Hispanic Black and Hispanic cancer survivors were 1.79 (1.32, 2.34), and 2.03 (1.61, 252) higher odds of reporting fair-to-poor mental health compared to non-Hispanic White cancer survivors (Table 4).

**Table 4:** Models showing the contributing effect of discrimination on race/ethnicity disparities in health-related quality of life of cancer survivors from the All of Us

Discrimination in medical setting (DMS)							
Fair-to-poor physical health							
		Model 1 (*n* = 13,822)	Model 2 (*n* = 13,822)		% change	Model 3 (*n* = 13,555)	
	OR	95%CI	OR	95%CI		OR	95%CI
Asian	1.45	(0.79–2.44)	1.41	(0.77–2.41)	− 2.76	1.07	(0.55–1.95)
Black	2.48	(1.89–3.20)***	2.15	(1.63–2.81)***	− 13.31	0.97	(0.71–1.32)
Hispanic	2.47	(1.98–3.07)***	2.32	(1.84–2.89)***	− 6.07	1.11	(0.84–1.45)
Other	2.53	(1.42–4.24)***	2.22	(1.23–3.76)**	− 12.25	1.60	(0.82–2.94)
White	Ref						
Fair-to-poor mental health							
		Model 1 (*n* = 10,552)	Model 2 (*n* = 10,552)			Model 3 (*n* = 10,322)	
	OR	95%CI	OR	95%CI		OR	95%CI
Asian	0.69	(0.31–1.34)	0.69	(0.31–1.34)	0.00	0.47	(0.20–0.97)*
Black	1.78	(1.32–2.34)***	1.53	(1.14–2.04)**	− 14.04	0.74	(0.54–1.02)
Hispanic	2.03	(1.61–2.52)***	1.98	(1.57–2.48)***	− 2.46	1.04	(0.79–1.36)
Other	1.54	(0.79–2.74)	1.38	(0.70–2.48)	− 10.39	1.02	(0.50–1.94)
White	Ref						
Perceived discrimination (PD)							
Fair-to-poor physical health							
		Model 1 (*n* = 13,822)	Model 2 (*n* = 13,822)			Model 3 (*n* = 13,555)	
	OR	95%CI	OR	95%CI		OR	95%CI
Asian	1.45	(0.79–2.44)	1.23	(0.67–2.10)	− 15.17	1.01	(0.52–1.82)
Black	2.48	(1.89–3.20)***	1.73	(1.30–2.64)***	− 30.24	0.91	(0.66–1.23)
Hispanic	2.47	(1.98–3.07)***	1.96	(1.55–2.46)***	− 20.65	1.05	(0.79–1.37)
Other	2.53	(1.42–4.24)***	1.83	(1.01–3.11)*	− 27.67	1.49	(0.76–2.73)
White	Ref						
Fair-to-poor mental health							
		Model 1 (*n* = 10,552)	Model 2 (*n* = 10,552)			Model 3 (*n* = 10,322)	
	OR	95%CI	OR	95%CI		OR	95%CI
Asian	0.69	(0.31–1.34)	0.59	(0.26–1.15)	− 14.49	0.43	(0.18–0.89)*
Black	1.78	(1.32–2.34)***	1.22	(0.90–1.64)	− 31.46	0.67	(0.48–0.92)*
Hispanic	2.03	(1.61–2.52)***	1.76	(1.38–2.20)***	− 13.30	0.99	(0.75–1.29)
Other	1.54	(0.79–2.74)	1.19	(0.60–2.15)	− 22.73	0.95	(0.47–1.81)
White	Ref						

*Notes*: Model 1 = race/ethnicity and outcome, model 2 = model 1 + DMS or PD, model 3 = model 2 + covariates: sex, age, marital status, active treatment, SES barriers, nativity & cancer type

% change = percent change in the odds from model 1 and model 2

*Ref*, reference group; *CI*, confidence interval, significant *p*-values *** < 0.001, ** < 0.01, * < 0.05

### Race/Ethnicity and Physical Health—Adjusted for DMS or PD (Model 2)

Adding DMS or PD to our model 1, we found that by adding DMS in model 1, a decrease in the odds of fair-to-poor physical health was observed for non-Hispanic Black (− 13.3%) and Hispanic (− 6.1%) cancer survivors compared to non-Hispanic White cancer survivors. While controlling for PD, a decrease in the odds of reporting fair-to-poor physical health was found for non-Hispanic Black (− 30.2%) and Hispanic (− 20.7%) cancer survivors compared to non-Hispanic White cancer survivors (Table 4).

### Race/Ethnicity and Mental Health—Adjusted for DMS or PD (Model 2)

Adding DMS to our model 1, results showed a 14% and 2.5% decrease in the odds of reporting fair-to-poor mental health for non-Hispanic Black and Hispanic survivors, respectively whereas, after controlling for PD in our model, the odds of reporting fair-to-poor mental health decreased by 31% and 13.3% for non-Hispanic Black and Hispanic compared to non-Hispanic White cancer survivors, respectively (Table 4).

### Race/Ethnicity and HRQoL—Adjusted for DMS or PD and Covariates (Model 3)

Our last multivariable models with DMS or PD plus covariates showed no racial/ethnic disparities in the likelihood of fair-to-poor physical health (Table 4, model 3). However, for PD, we found that compared to non-Hispanic White cancer, non-Hispanic Black cancer survivors had 0.67 (0.48, 0.92) lower odds of reporting fair-to-poor mental health. We found that overall in our cohort, non-Hispanic Asian cancer survivors reported lower odds to report fair-to-poor physical and mental health compared to non-Hispanic White cancer survivors after adjusting for DMS or PD and covariates [fair-to-poor physical health: 0.47 (0.20, 0.94); fair-to-poor mental health 0.43 (0.18, 0.89)].

## Discussion

Using data from the All of Us research cohort, we assessed the association between discrimination (DMS and PD) and health-related quality of life (HRQoL) among cancer survivors while exploring their potential roles in lessening racial/ethnic disparities in HRQoL. After adjusting for covariates, experiencing discrimination in the medical settings and perceived were independently associated with increased odds of fair-to-poor HRQoL. We observed that disparities in HRQoL between non-Hispanic Black and non-Hispanic White cancer survivors and between Hispanic and non-Hispanic White cancer survivors were attenuated after controlling for exposure to discrimination. The attenuation was greater among non-Hispanic Black cancer survivors.

Our findings mirror those in other populations in the US, where those who reported experiencing discrimination tended to score lower for HRQoL [42, 43], and the effects are worse among non-Hispanic Blacks and Hispanic populations [44–47]. Only a few studies have investigated the effect of discrimination experiences among cancer survivors and their results are similar to the ones found in this study. For example, among breast cancer survivors, exposure to racial/ethnic discrimination had a negative impact on the HRQoL of survivors [6]. Karvonen and colleagues also found that non-Hispanic Black, Hispanic, and other racial/ethnic minoritized cancer survivors were more likely to report worse physical and mental health outcomes compared to those who did not experience discrimination [48]. Similarly, Shariff-Marco and colleagues found that among non-Hispanic Black and Hispanic cancer survivors who reside in less diverse areas, exposure to racial discrimination was associated with lower HRQoL [6]. Our study, however, builds on this work because we demonstrate that eliminating exposure to discrimination in the healthcare setting (e.g., via implicit/explicit bias and cultural competence training, and engaging with community members in the areas they serve) can potentially attenuate racial/ethnic disparities in HRQoL among cancer survivors.

Our study expands discrimination research among cancer survivors by exploring the impact of everyday PD and DMS. Nonetheless, our findings are similar to other studies have reported [49, 50]. Survivors may interact with medical personnel and institutions more than the general population. Our results showed that when we controlled for everyday PD, the likelihood of reporting fair-to-poor mental health for non-Hispanic Black cancer survivors was no longer significant compared to non-Hispanic White. This suggests that disparities in the mental health of non-Hispanic Black cancer survivors can be lessened if we address PD. Furthermore, the negative interpersonal and systemic interactions encountered by these cancer survivors can have a downstream effect that may lead to a decrease in and delay the healthcare needs [21] of non-Hispanic Black and Hispanic cancer survivors, by addressing discrimination, a potential improvement in health-related quality of life may be achieved for all cancer survivors.

### Strengths, Future Direction, and Limitations

Using of data from the All of Us research program allows for a large sample of cancer survivors. Given that the All of Us research currently continues enrolling participants and current enrollees have the option to continue answering surveys (i.e., SDoH survey), this study can be replicated to determine if our findings remain consistent. Future studies should explore variation across race/ethnicities among non-Hispanic Black and Hispanics, as these are heterogeneous and vary across cultural and SES aspects. For example, within the Hispanic population, evidence suggests that some groups may experience more discrimination or be perceived as criminal or second-class citizens (Mexicans and Puerto Ricans), whereas Cubans may have been favored by policies implemented by the US government [51], thus possibly experiencing fewer discriminatory events affecting their association with their HRQoL.

Our study is not without limitations. While using this dataset is a strength of this study, it also limits our generalizability as the All of Us research program is not meant to be a US population-based cohort. The cross-sectional design limits establishing temporal or causal relationships. However, being the first to assess the relationship between DMS and HRQoL among this population provides a future direction in research by allowing comparisons to other cancer survivor populations. Our sample was predominantly comprised of a higher distribution of cancer survivors with a college degree or higher, and higher income status, thus limiting our generalizability. Additionally, those who answered the SDoH survey were less likely to be of race/ethnicity minoritized groups, potentially introducing participation bias. However, this bias could be a non-differential driving our findings towards the null given that we are not sure whether those who did not complete the survey experienced less or more discriminatory experiences or reported worse or better HRQoL. We were also not able to control for comorbidities; however, to minimize residual confounding of comorbidities in our models, we controlled for current cancer treatment status, as it was previously suggested that comorbidities and disease status were factors identified by survivors who reported greater discrimination experiences. Similarly, we were not able to control for residing in an ethnic enclave, especially because there is evidence suggesting that exposure to racial discrimination was linked to lower HRQoL among cancer survivors living in less diverse areas [6]. Thus, future studies should account for living in an ethnic enclave and socioeconomic neighborhood attributes (e.g., green spaces, food, and pharmacy deserts). Lastly, we are not able to account for treatment type or disease severity as these factors can impact the HRQoL of cancer survivors.

### Implication

There has been a push in the US, to create national awareness of the adverse health outcomes among ethnic minoritized individuals. In addition to awareness, attempts to promote equity and mitigate health disparities by tackling structural and institutional racial discrimination have been supported [52, 53]. Findings from this study can help guide clinicians and policymakers to continue to lessen the impact of discrimination on the HRQoL among cancer survivors by creating protocols and policies at the health system and government levels to promote inclusivity. For example, in New Zealand, nursing practices have incorporated the Moari practice model to guide healthcare centers in providing better care for this population [54]. This model perhaps served as an interpersonal-level policy and protocol that incorporates cultural components to promote a better understanding of this diverse group. By implementing such policies and protocols, discriminatory practices in medical settings can potentially be reduced as medical professionals have a better cultural understanding of these diverse groups, promoting less discriminatory events, and ultimately leading to better health outcomes for racial/ethnic minoritized groups.

## Conclusion

Our study underscores that discrimination (perceived in medical settings and everyday) contributes to the racial/ethnic disparities in reporting fair-to-poor HRQoL among non-Hispanic Black and Hispanic cancer survivors compared to non-Hispanic White cancer survivors from the All of Us cohort. The greatest impact in lessening the effects of reporting fair-to-poor HRQoL was seen for non-Hispanic Black cancer survivors. More importantly, it suggested that if we only mitigate PD, disparities in mental health are no longer seen for non-Hispanic Black cancer survivors compared to non-Hispanic White cancer survivors. Future research using longitudinal data is needed to continue to assess the association of discrimination experiences in the medical setting and everyday with the HRQoL of cancer survivors to highlight the disparities experienced by these historically marginalized populations.

## Supplementary Information

Below is the link to the electronic supplementary material.

Supplementary file1 (DOCX 22 KB)

## Data Availability

This study used data from the All of Us data resource. The interpretation and reporting of these data are the sole responsibility of the authors. The data is publicly available with approval for use from the NIH All of Us research program on the workbench.

## Code Availability

For code, please email the corresponding author.
