# Supplementary material for: Racial/Ethnic Disparities: Discrimination’s Impact on Health-Related Quality of Life—An All of Us Cancer Survivors’ Cross-sectional Study
Source: J Racial Ethn Health Disparities. 2024 Apr 23;12(3):1765–76. doi: 10.1007/s40615-024-02006-z (PMC11496381; doi:10.1007/s40615-024-02006-z)
Supplement: Supplementary file 1 — Supplementary file1 (DOCX 22 KB) [file 40615_2024_2006_MOESM1_ESM.docx]

| Supplemental Table 1: Definition and Classification of Discrimination in Medical Settings (DMS) | |
| --- | --- |
| Item Description | **Response Options** |
| 1. You are treated with less courtesy than other people | 0 – Never, 1 – Rarely, 2 – Sometimes, 3 – Most of the time, 4 – Always |
| 1. You are treated with less respect than other people |  |
| 1. You receive poorer service than others. |  |
| 1. A doctor or nurse acts if he or she thinks you are not smart. |  |
| 1. A doctor or nurse acts as if he or she is afraid of you. |  |
| 1. A doctor or nurse acts as if he or she is better than you. |  |
| 1. You feel like a doctor or nurse is not listening to what you were saying. |  |
| DMS composite Score | |
| Composite score of the sum of DMS items 1-7 | Range [Min: 0 – Max:28] |
| Scaled composite DMS score by 5 units increments | Range [Min: 0 – Max:5.6] |

Supplementary Tables

| Supplemental Table 2: Definition and Classification of Everyday Perceived Discrimination (PD) | |
| --- | --- |
| Item Description | **Response Options** |
| 1. You are treated with less courtesy than other people are. | 0 – Never, 1 – Less than once a year, 2 – A few times a year, 3 – A few times a month, 4 – At least once a week, 5 – Almost every day |
| 1. You are treated with less respect than other people are. |  |
| 1. You receive poorer service than other people at restaurants or stores. |  |
| 1. People act as if they think you are not smart. |  |
| 1. People act as if they are afraid of you. |  |
| 1. People act as if they think you are dishonest. |  |
| 1. People act as if they’re better than you are. |  |
| 1. You are called names or insulted. |  |
| 1. You are threatened or harassed. |  |
| PD composite Score | |
| Composite score of the sum of PD items 1-9 | Range [Min: 0 – Max:45] |
| Scaled composite PD score by 5 units increments | Range [Min: 0 – Max:9] |

| Supplemental Table 3: Definition and Classification of Health-Related Quality of Life (HRQoL) | |
| --- | --- |
| Items Description | |
| Physical Health(PH) | |
| 1. In general, how would you rate your physical health? | |
| Response options: 1= Poor, 2= Fair, 3= Good, 4= Very good, 5= Excellent | |
| 1. To what extent are you able to carry out your everyday physical activities such as walking, climbing stairs, carrying groceries, or moving a chair? | |
| Response options: 1= Not at all, 2= A little, 3= Moderately, 4= Mostly , 5= Completely | |
| 1. In the past 7 days, how would you rate (scale of 0 – 10) your pain on average? | |
| Response options: 1= 10, 2=(9,8,7), 3=(6,5,4), 4=(3,2,1), 5= 0 | |
| 1. In the past 7 days, how would you rate your fatigue? | |
| Response options: 1= Very severe, 2= Severe, 3= Moderate, 4= Mild , 5= None | |
| Physical Health Composite | |
| PH raw score: composed of PROMIS subscale items 1-4 | Range [Min:4 – Max:20] |
| PH T-score: based on PH raw score. | Range [Min:16.2 – Max:67.7] |
| Dichotomous composite indicator |  |
| < 42 (fair-to-poor) | Low physical health(PH) |
| ≥ 42(good-to-excellent) | High physical health |
|  |  |
| Mental Health (MH) | |
| 1. In general, how would you say your quality of life is? | |
| Response options: 1= Poor, 2= Fair, 3= Good, 4= Very good, 5= Excellent | |
| 1. In general, how would you rate your mental health, including your mood and your ability to think? | |
| Response options: 1= Poor, 2= Fair, 3= Good, 4= Very good, 5= Excellent | |
| 1. In general, how would you rate your satisfaction with your social activities and relationships? | |
| Response options: 1= Poor, 2= Fair, 3= Good, 4= Very good, 5= Excellent | |
| 1. In the past 7 days, how often have you been bothered by emotional problems such as feeling anxious depressed or irritable? | |
| Response options: 1= Always, 2= Often, 3= Sometimes, 4= Rarely , 5= Never | |
| Mental Health Composite | |
| MH raw score: composed of PROMIS subscale items 5-8 | Range [Min:4 – Max:20] |
| MH T-score: based on MH raw score. | Range [Min:21.2 – Max:67.6] |
| Dichotomous composite indicator |  |
| < 40 (fair-to-poor) | Low physical health(PH) |
| ≥ 40(good-to-excellent) | High physical health |
