# Supplementary material for: Racial/Ethnic Disparities: Discrimination’s Impact on Health-Related Quality of Life—An All of Us Cancer Survivors’ Cross-sectional Study
Source: J Racial Ethn Health Disparities. Author manuscript; Available in PMC 2025 Jun 1. (PMC11496381; doi:10.1007/s40615-024-02006-z)
Supplement: Supplementary file 1 [file NIHMS1988679-supplement-Supplementary_tables.docx]

| Supplemental Table 1: Definition and Classification of Discrimination in Medical Settings (DMS) |  |
| --- | --- |
| Item Description | **Response Options** |
| 1) You are treated with less courtesy than other people | 0 – Never, 1 – Rarely, 2 – Sometimes, 3 – Most of the time, 4 – Always |
| 2) You are treated with less respect than other people |  |
| 3) You receive poorer service than others. |  |
| 4) A doctor or nurse acts if he or she thinks you are not smart. |  |
| 5) A doctor or nurse acts as if he or she is afraid of you. |  |
| 6) A doctor or nurse acts as if he or she is better than you. |  |
| 7) You feel like a doctor or nurse is not listening to what you were saying. |  |
| DMS composite Score |  |
| Composite score of the sum of DMS items 1-7 | Range [Min: 0 – Max:28] |
| Scaled composite DMS score by 5 units increments | Range [Min: 0 – Max:5.6] |

Supplementary Tables

| Supplemental Table 2: Definition and Classification of Everyday Perceived  Discrimination (PD) |  |
| --- | --- |
| Item Description | **Response Options** |
| 1) You are treated with less courtesy than other people are. | 0 – Never, 1 – Less than once a year, 2 – A few times a year, 3 – A few times a month, 4 – At least once a week, 5 – Almost every day |
| 2) You are treated with less respect than other people are. |  |
| 3) You receive poorer service than other people at restaurants or stores. |  |
| 4) People act as if they think you are not smart. |  |
| 5) People act as if they are afraid of you. |  |
| 6) People act as if they think you are dishonest. |  |
| 7) People act as if they’re better than you are. |  |
| 8) You are called names or insulted. |  |
| 9) You are threatened or harassed. |  |
| PD composite Score |  |
| Composite score of the sum of PD items 1-9 | Range [Min: 0 – Max:45] |
| Scaled composite PD score by 5 units increments | Range [Min: 0 – Max:9] |

| Supplemental Table 3: Definition and Classification of Health-Related Quality of Life (HRQoL) |  |
| --- | --- |
| Items Description |  |
| Physical Health(PH) |  |
| 1) In general, how would you rate your physical health? |  |
| Response options: 1= Poor, 2= Fair, 3= Good,  4= Very good, 5= Excellent |  |
| 2) To what extent are you able to carry out your everyday physical activities such as walking, climbing stairs, carrying groceries, or moving a chair? |  |
| Response options: 1= Not at all, 2= A little, 3= Moderately,  4= Mostly , 5= Completely |  |
| 3) In the past 7 days, how would you rate (scale of 0 – 10) your pain on average? |  |
| Response options: 1= 10, 2=(9,8,7), 3=(6,5,4), 4=(3,2,1), 5= 0 |  |
| 4) In the past 7 days, how would you rate your fatigue? |  |
| Response options: 1= Very severe, 2= Severe, 3= Moderate,  4= Mild , 5= None |  |
| Physical Health Composite |  |
| PH raw score: composed of PROMIS subscale items 1-4 | Range [Min:4 – Max:20] |
| PH T-score: based on PH raw score. | Range [Min:16.2 – Max:67.7] |
| Dichotomous composite indicator |  |
| < 42 (fair-to-poor) | Low physical health(PH) |
| ≥ 42(good-to-excellent) | High physical health |
|  |  |
| Mental Health (MH) |  |
| 5) In general, how would you say your quality of life is? |  |
| Response options: 1= Poor, 2= Fair, 3= Good,  4= Very good, 5= Excellent |  |
| 6) In general, how would you rate your mental health, including your mood and your ability to think? |  |
| Response options: 1= Poor, 2= Fair, 3= Good,  4= Very good, 5= Excellent |  |
| 7) In general, how would you rate your satisfaction with your social activities and relationships? |  |
| Response options: 1= Poor, 2= Fair, 3= Good,  4= Very good, 5= Excellent |  |
| 8) In the past 7 days, how often have you been bothered by emotional problems such as feeling anxious depressed or irritable? |  |
| Response options: 1= Always, 2= Often, 3= Sometimes,  4= Rarely , 5= Never |  |
| Mental Health Composite |  |
| MH raw score: composed of PROMIS subscale items 5-8 | Range [Min:4 – Max:20] |
| MH T-score: based on MH raw score. | Range [Min:21.2 – Max:67.6] |
| Dichotomous composite indicator |  |
| < 40 (fair-to-poor) | Low physical health(PH) |
| ≥ 40(good-to-excellent) | High physical health |
